# Flagella of *Aeromonas veronii* biotype sobria promote biofilm formation by biofilm-derived outer membrane vesicles (bOMVs)

**DOI:** 10.1128/spectrum.02838-24

**Published:** 2025-10-27

**Authors:** Soshi Seike, Hidetomo Kobayashi, Eizo Takahashi, Keinosuke Okamoto, Hiroyasu Yamanaka

**Affiliations:** 1 Laboratory of Molecular Microbiological Science, Faculty of Pharmaceutical Sciences, Hiroshima International University 26281 , Kure, Hiroshima, Japan; 2 Laboratory of Medical Microbiology, Department of Health Pharmacy, Yokohama University of Pharmacy 68348, Yokohama, Kanagawa, Japan; 3 Graduate School of Medicine, Dentistry and Pharmaceutical Sciences, Okayama University 12997, Kita-ku, Okayama, Japan; University of Florida, Gainesville, Florida, USA

**Keywords:** *Aeromonas veronii* biotype sobria, flagella, outer membrane vesicles (OMVs), biofilm

## Abstract

*Aeromonas veronii* biotype sobria is a gram-negative rod-shaped bacterium commonly found in various water sources. *A. veronii* sobria can form biofilms and has been implicated in sporadic infections associated with medical devices like catheters. Previous studies have noted that biofilm-derived outer membrane vesicles (bOMVs) can enhance biofilm formation, though the impact varies across different strains. It remains uncertain whether these variances are attributable to bacterial characteristics or bOMVs components. To investigate this, we analyzed the effects of bOMVs using three typical *A. veronii* sobria strains (102, 104, and 106) with varying biofilm-forming abilities, noting disparities in biofilm formation by bOMVs derived from each strain. Detailed focus on strain 106, the most responsive to bOMVs, led to the discovery of distinct characteristics, including the presence of flagella-like structures and their role in biofilm formation.

**IMPORTANCE:**

This study illuminates the role of *A. veronii* biotype sobria flagella in promoting biofilm formation by bOMVs through diverse mechanisms. The findings suggest a significant interaction between flagella, outer membrane vesicles, and bacterial cells, influencing the biofilm development process. Understanding these mechanisms could provide crucial insights into the pathogenic potential of *A. veronii* biotype sobria strains and potentially inform novel strategies for combating biofilm-related infections.

## INTRODUCTION

*Aeromonas veronii* biotype sobria is a gram-negative, facultative anaerobic rod commonly found in diverse water sources. *A. veronii* sobria is a known human pathogen that causes foodborne illnesses but demonstrates opportunistic behavior, manifesting especially in immunocompromised and elderly patients (1). It can also cause serious extraintestinal conditions such as sepsis, peritonitis, and meningitis (2). Because this bacterium multiplies rapidly after infection and the infectious disease progresses quickly, it is considered particularly important to take effective infection control measures for these patients.

Outer membrane vesicles (OMVs) are microvesicles released by gram-negative bacteria and exhibit diverse biological activities. Various functions have been reported in bacterial interactions, including biofilm formation (3–5), antibactericidal activity (6), toxin delivery (7), and gene transport (8, 9). We previously documented the enhancement of biofilm formation by biofilm-derived outer membrane vesicles (bOMVs), originating from bacterial biofilms in *Aeromonas* spp. (4). Similar findings have also been reported in other bacterial species, including *Helicobacter pylori*, *Pseudomonas aeruginosa*, and *Vibrio cholerae* (4, 10–12). Nonetheless, the precise mechanisms by which OMVs, particularly bOMVs, contribute to bacterial biofilm formation remain unclear.

*Aeromonas* are essentially known as flagellated bacteria (13, 14), and *Aeromonas hydrophila* has been reported to have sodium-driven motility, similar to *Vibrio* spp. (15). *A. veronii* sobria has also been reported to have flagella, but no data on their driving force have yet been presented (16). In *Aeromonas* spp. and other bacteria (*Escherichia coli*, *Pseudomonas aeruginosa*,* Vibrio alginolyticus*, etc.), flagella are thought to contribute to bacterial motility and to adhesion (17–19). In addition, it has been reported that the rotation of flagella promotes the production of OMVs in the genus *Vibrio fischeri* (20). Based on the above findings, we hypothesized that the flagella and bOMVs may also work cooperatively in *A. veronii* sobria.

In this study, three strains of *A. veronii* sobria with different biofilm formation capacities were used to examine differences in bOMVs ability to promote biofilm formation. As a result, a strain susceptible to biofilm formation by bOMVs from other strains was identified. Unlike the other two strains, this strain had flagella. Therefore, we hypothesized that the flagella might affect the interaction between bOMVs and the bacteria. To verify this hypothesis, we investigated the relationship between the flagella and the biofilm formation-promoting effect of bOMVs. As a result, we found that the flagella of bOMVs of other bacterial strains interact with bOMVs and that the contact frequency between the bacteria and bOMVs increases due to the movement of the bacteria through the flagella. In this paper, we discuss how this phenomenon plays a role in the biofilm formation of this bacterium.

## RESULTS

### Biofilm-forming ability varies among *A. veronii* sobria strains

**Fig 1:**
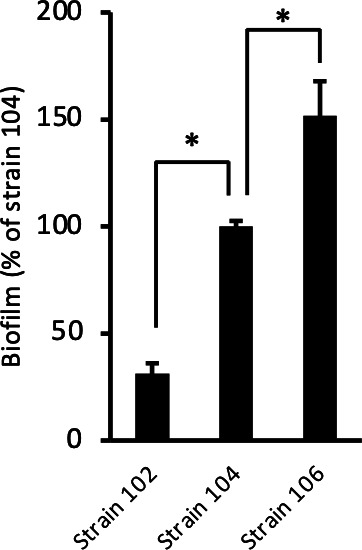
Biofilm-forming ability varies among *A. veronii* sobria strains. Biofilm formation on 96-well flat-bottom polystyrene plates was determined by our previous method (4) using crystal violet. The values in the graph are shown as relative values when the biofilm-forming ability of *A. veronii* sobria strain 104 was set at 100%. Experiments were performed in triplicate on two independent occasions. Data are shown as the mean and standard error. * indicates a significant difference at *P* < 0.05.

In our previous study, we observed differences in the biofilm-forming abilities of various *Aeromonas* strains through *in vitro* analysis (4). In this study, we used three strains of *A. veronii* sobria (102, 104, and 106) to examine biofilm-forming ability; these strains were isolated from different diarrhea patients at the same medical facilities. The biofilm-forming ability of strain 104, classified as moderate, was set at 100%, and the abilities of the other two strains were compared against it. As shown in Fig. 1, the biofilm-forming abilities of strains 106 and 102 were higher and lower, respectively, than that of strain 104.

### Variability in the impact of bOMVs on *A. veronii* sobria biofilm formation across different strains

**Fig 2:**
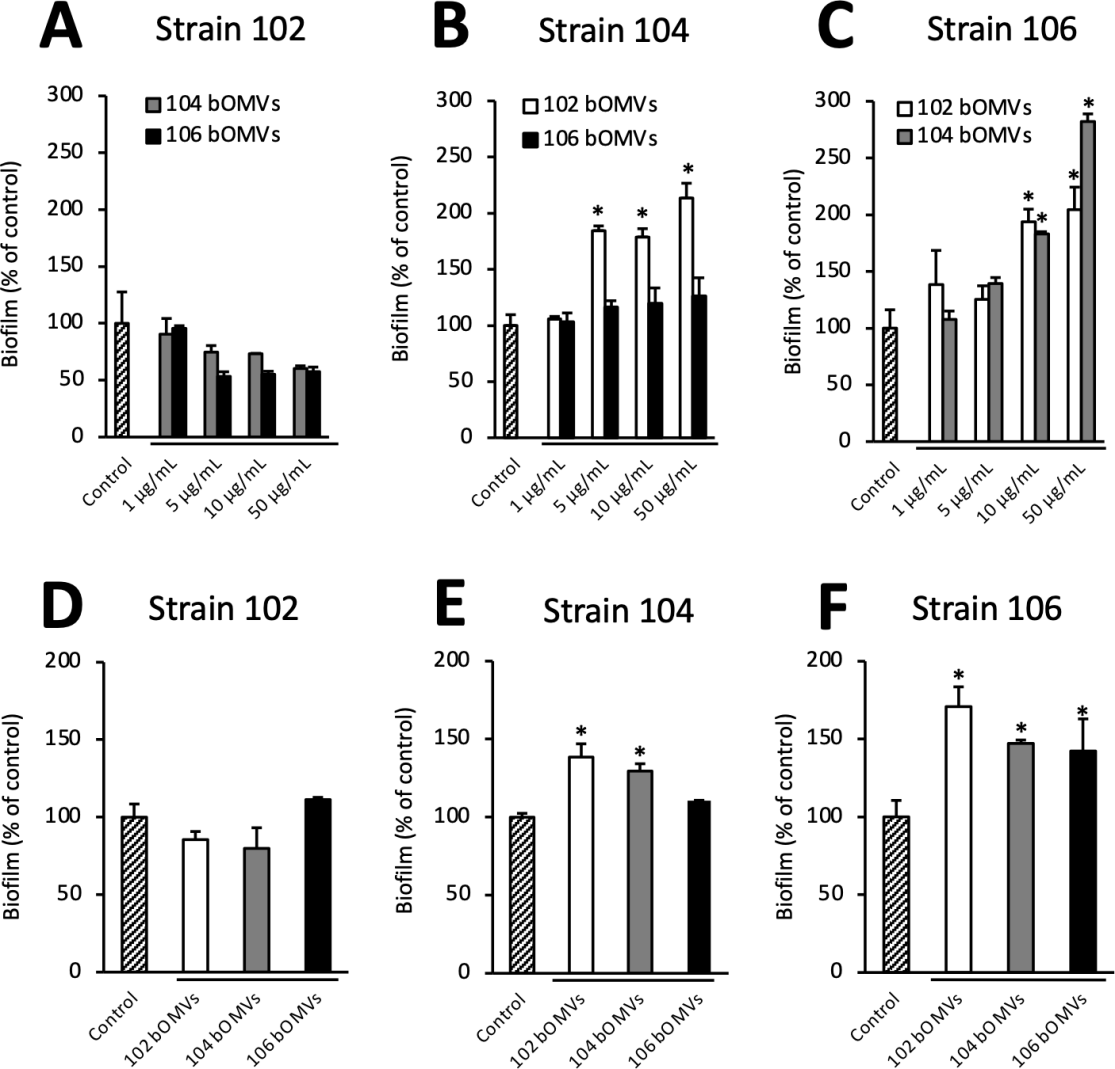
Variability in the impact of bOMVs on *Aeromonas* biofilm formation across different strains. Biofilm-forming abilities of *A. veronii* sobria strains 102, 104, and 106 were measured in the presence of purified 104 or 106 bOMVs (**A**), 102 or 106 bOMVs (**B**), and 102 or 104 bOMVs (**C**), respectively. Purified bOMVs were added at the indicated concentrations. To clarify the differences in biofilm-forming ability that appear when a fixed amount of bOMVs is added to all strains used, the biofilm-forming ability of each *A. veronii* sobria strain in the presence of 10 µg/mL of each purified bOMVs was also compared (**D**–**F**). Experiments were performed in triplicate on two independent occasions. Data are shown as the mean and standard error. * indicates a significant difference at *P* < 0.05 compared to the control experiment.

We next investigated the factors underlying the differences in biofilm-forming ability observed among *A. veronii* sobria strains. Following our earlier discovery that bOMVs released by a particular bacterial strain enhance the biofilm formation of that same strain (4), here we investigated whether bOMVs derived from a different strain could affect the biofilm-forming ability of each strain. Since phosphate-buffered saline (PBS) was used as the suspension solution for purified bOMVs, an experimental sample with only PBS served as a control, and its biofilm-forming ability was compared to that of the sample with purified bOMVs. The results demonstrated that the biofilm-forming ability of *A. veronii* sobria strain 102 did not change significantly even when purified bOMVs derived from strain 104 (104 bOMVs) or 106 (106 bOMVs) were added (Fig. 2A). The biofilm-forming ability of strain 104 was clearly increased by the addition of purified 102 bOMVs, while the addition of purified 106 bOMVs increased it slightly but not significantly (Fig. 2B). In contrast, the biofilm-forming ability of strain 106 was increased in a dose-dependent manner by the addition of either purified 102 bOMVs or 104 bOMVs (Fig. 2C).

To compare the effect of purified bOMVs on the biofilm-forming ability of each *A. veronii* sobria, we measured the changes in the biofilm-forming abilities of the three *A. veronii* sobria with the inclusion of purified bOMVs (10 µg/mL; the concentration of bOMVs was expressed as the protein concentration of the sample containing bOMVs). The results supported the findings described above; that is, the biofilm-forming ability of strain 102 was not altered by the addition of purified bOMVs from any of the three strains (Fig. 2D). The biofilm-forming ability of strain 104 was significantly increased by the addition of purified 102 bOMVs or 104 bOMVs. The addition of purified 106 bOMVs also slightly, but not significantly, increased the ability compared to the control (Fig. 2E). In contrast, the biofilm-forming ability of strain 106 increased significantly upon the addition of purified bOMVs from any of the three strains (Fig. 2F). Thus, the biofilm formation-promoting effects of bOMVs vary, depending on the strain of bacteria acting on them. This suggests that bOMVs-mediated biofilm formation may depend not only on the composition of the bOMVs but also on the characteristics of the strains.

### *Aeromonas veronii* sobria strain 106 had flagella-like structures on its bacterial surface

**Fig 3:**
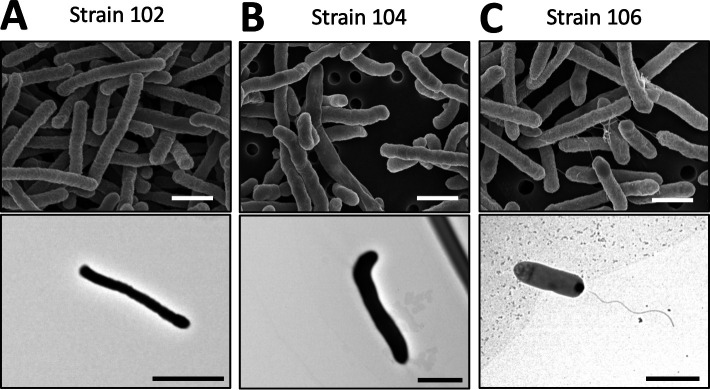
*Aeromonas veronii* sobria strain 106 had flagella-like structures on its bacterial surface. Scanning electron microscope and transmission electron microscope images of strains 102, 104, and 106 were shown in the panels **A**, **B,** and **C**, respectively. Strains 102 and 104 lack flagella, whereas flagella-like structures are clearly visible on the surface of strain 106. The white bar indicates a scale of 1 µm. The black bar indicates a scale of 2 µm.

To compare the surface structures of the *A. veronii* sobria strains used in this study, we observed those strains under a scanning electron microscope (SEM) and a transmission electron microscope (TEM). As shown in Fig. 3, we observed no flagellar or ciliated structures in strains 102 and 104 (Fig. 3A and B). In contrast, the cell surface of strain 106 (Fig. 3C) clearly showed many flagella-like structures compared to strains 102 and 104 (Fig. 3A and B). These flagella-like structures were entangled among the bacterial cells and flagella, suggesting an affinity for interacting with the bacterial body.

### Extracellular matrix fraction of *A. veronii* sobria strain 106 contained flagella-like structures in addition to bOMVs

**Fig 4:**
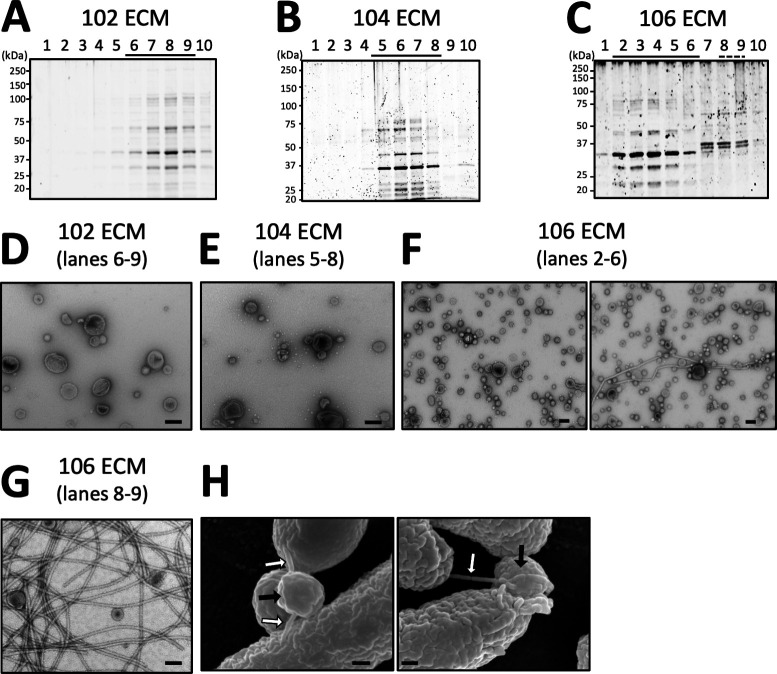
ECM fraction of *A. veronii* sobria strain 106 contained flagella-like structures, in addition to bOMVs. SDS-PAGE protein profiles of ECM preparations from *A. veronii* sobria 102 (**A**), 104 (**B**), and 106 (**C**) after fractionation by OptiPrep density gradient centrifugation. The fractionated ECM on these strains was analyzed by SDS-PAGE. The numbers in the figure correspond to the sample numbers obtained by OptiPrep density gradient centrifugation, with equal amounts taken from the lowest to highest density fractions. Lanes in which protein bands were identified (underlined lanes) were observed under a transmission electron microscope (TEM) after enrichment (**D–F**). TEM image of ECM lanes 8 and 9 prepared from strain 106 is shown in panel **G**. SEM image of strain 106 is also shown in panel H. The white arrows indicate flagella-like structures on the bacterial cell surface. The black arrows point to bOMVs released from the bacterial cells. The black bars correspond to a scale of 100 nm (**D**–**H**).

Since it has been reported that the extracellular matrix (ECM) of biofilms formed by flagellated bacteria contains flagella (21, 22), we fractionated the ECM of each of the three strains into 10 fractions using OptiPrep density gradient centrifugation to analyze the bacteria-derived structures within the ECM. Samples were prepared with the collected fractions and subjected to SDS-PAGE (Fig. 4A through C). The numbers in the figure correspond to the sample numbers obtained by OptiPrep density gradient centrifugation, with equal amounts taken from the lowest to highest density fractions. In the ECM fractions of strains 102 and 104, we identified limited protein bands, likely stemming from bOMVs, through SDS-PAGE analysis (lanes 6 to 9 in Fig. 4A and lanes 5 to 8 in Fig. 4B), and TEM observations revealed the exclusive presence of bOMVs in these samples (Fig. 4D and E). In contrast, similar protein bands thought to be derived from bOMVs were also detected in the ECM fraction of strain 106 by SDS-PAGE (Fig. 4C, lanes 2 to 6). Observation through TEM revealed the presence of bOMVs in most of these samples (Fig. 4F, left panel). However, in a few fields of view of the panels taken, we also detected the presence of flagella-like structures, with some observed to attach to bOMVs (Fig. 4F, right panel). The SDS-PAGE profile showed that the high-density OptiPrep fraction contained protein bands distinct from those originating from bOMVs (Fig. 4C, lanes 8 and 9). Subsequent TEM examination of lanes 8 and 9 clearly revealed flagella-like structures, some of which appeared to bind to bOMVs (Fig. 4G). In fact, SEM observation of the 106 strains cultured in liquid medium showed the binding of flagella-like structures and OMV-like structures, as shown in Fig. 4H.

### The proteins found in the high-density OptiPrep fraction of the ECM from *A. veronii* sobria strain 106 were identified as flagellin

**Fig 5:**
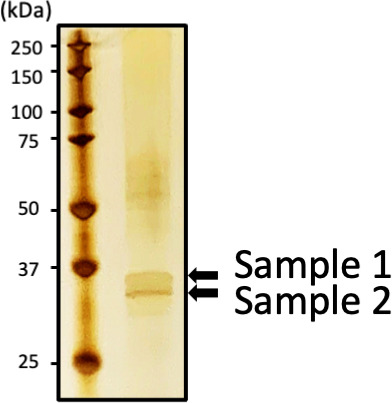
SDS-PAGE profile of proteins constituting flagella-like structures of strain 106. Two clearly identified protein bands were extracted from the gel and designated as samples 1 and 2. These samples were subjected to nano-LCMS/MS analysis.

To identify the flagella-like structures found in the ECM fraction of strain 106, flagella-like structures were purified from fractions 8 to 9, as illustrated in Fig. 4C. After the purified sample was fractionated by SDS-PAGE, we excised the two primary protein bands from the gel after SDS-PAGE (Fig. 5, samples 1 and 2), retrieving the proteins contained in the gel. The proteins in each excised gel band were then identified by nano-liquid chromatography-tandem mass spectrometry (LCMS/MS) analysis.

As shown in Table 1, the results clearly suggested that the two primary proteins extracted from the gel (both samples 1 and 2) were flagellins, which constitute the polar flagella.

**TABLE 1:** Identification of proteins extracted from SDS-PAGE gel by nano-LCMS/MS analysis

Results									
Sample label		Protein	Gene	MW	Score	Peptide	Coverage	Accession	Note^*a*^
Sample 1	1	Flagellin A		32,229	334	11	36		BCT36488.1
	2	Flagellin B		32,141	257	12	39		WP_118853647.1
	3	Flagellin	flaA	32,287	220	9	28	B3TZ71	ACB38418.1
Sample 2	1	Flagellin A		32,229	417	17	38		BCT36488.1
	2	Flagellin B		32,141	203	13	27		WP_118853647.1
	3	Mu-like prophage major head subunit gpT family protein		33,501	92	5	12		WP_042084095.1

^*a*^ Accession in Mascot search results.

### The interaction between bOMVs and flagella varies, depending on the strain from which the bOMVs originate

From the experimental results outlined above, it is suggested that the presence of flagella on the bacterial surface of strain 106 may enhance the interaction with bOMVs. We therefore focused on this consideration in our further investigation.

**Fig 6:**
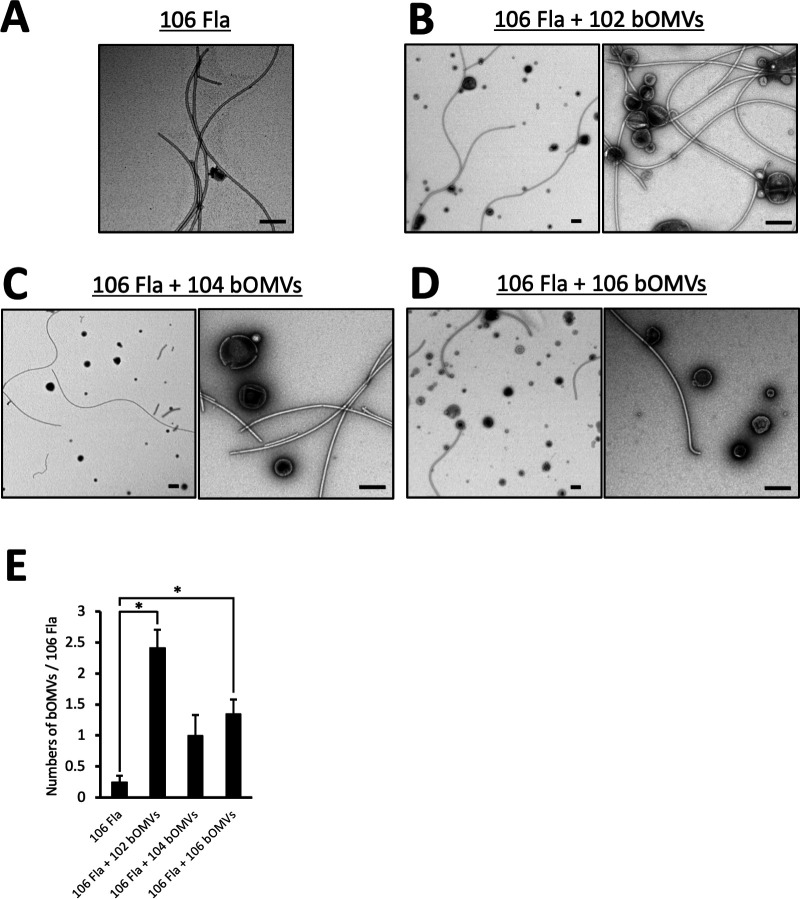
The interaction between bOMVs and flagella varies, depending on the strain from which the bOMVs originate. Purified 106 Fla (106 Fla) (**A**) was mixed with purified 102 bOMVs (**B**), 104 bOMVs (**C**), and 106 bOMVs (**D**). After reacting at 30°C for 2 h, the mixture was observed by TEM. The black bars correspond to a scale of 200 nm. (**E**) Four fields of view of ×5,000 images taken under reaction conditions **A–D **were used to count and graph the number of bOMVs bound to one 106 Fla. Data are shown as the mean and standard error. * indicates a significant difference at *P* < 0.05.

To examine the direct interaction between flagella and bOMVs, purified bOMVs were added to flagella, and their interaction was observed by TEM. As shown in Fig. 6A, the experiment was carried out after confirming that the flagella preparation derived from strain 106 (106 Fla) did not contain bOMVs released by the bacterial cells themselves. In the experiments shown in Fig. 4D through F, we observed bOMVs with a protein concentration of 100 µg/mL. However, in areas where bOMVs were densely packed, they appeared to be adhering to each other. Therefore, in the experiment shown in Fig. 6B through D, we diluted the sample 1/20 (5 µg/mL) and performed microscopic observation while avoiding overcrowding of bOMVs as much as possible.

TEM observations showed that the interaction of 106 Fla and bOMVs varied based on the strain from which the bOMVs originated; that is, 106 Fla seemed to have a more pronounced interaction with purified 102 bOMVs (Fig. 6B). In contrast, the interaction between 106 Fla and bOMVs was significantly lower when purified 104 bOMVs were present (Fig. 6C). The level of interaction between 106 Fla and purified 106 bOMVs fell in between the two (Fig. 6D). To further quantify the TEM observations shown in Fig. 6A to D, we analyzed TEM images taken from four fields at a low magnification (×5,000) (Fig. S1). Quantification of the total number of bOMVs bound to flagella was correlated with the results of images observed by TEM as shown in Fig. 6E. These results indicate that the interaction of bOMVs with 106 Fla is altered by changing the origin of the bOMVs.

### Flagellar motility affects the interaction between bacterial cells and bOMVs and the bOMV’s ability to promote biofilm formation

**Fig 7:**
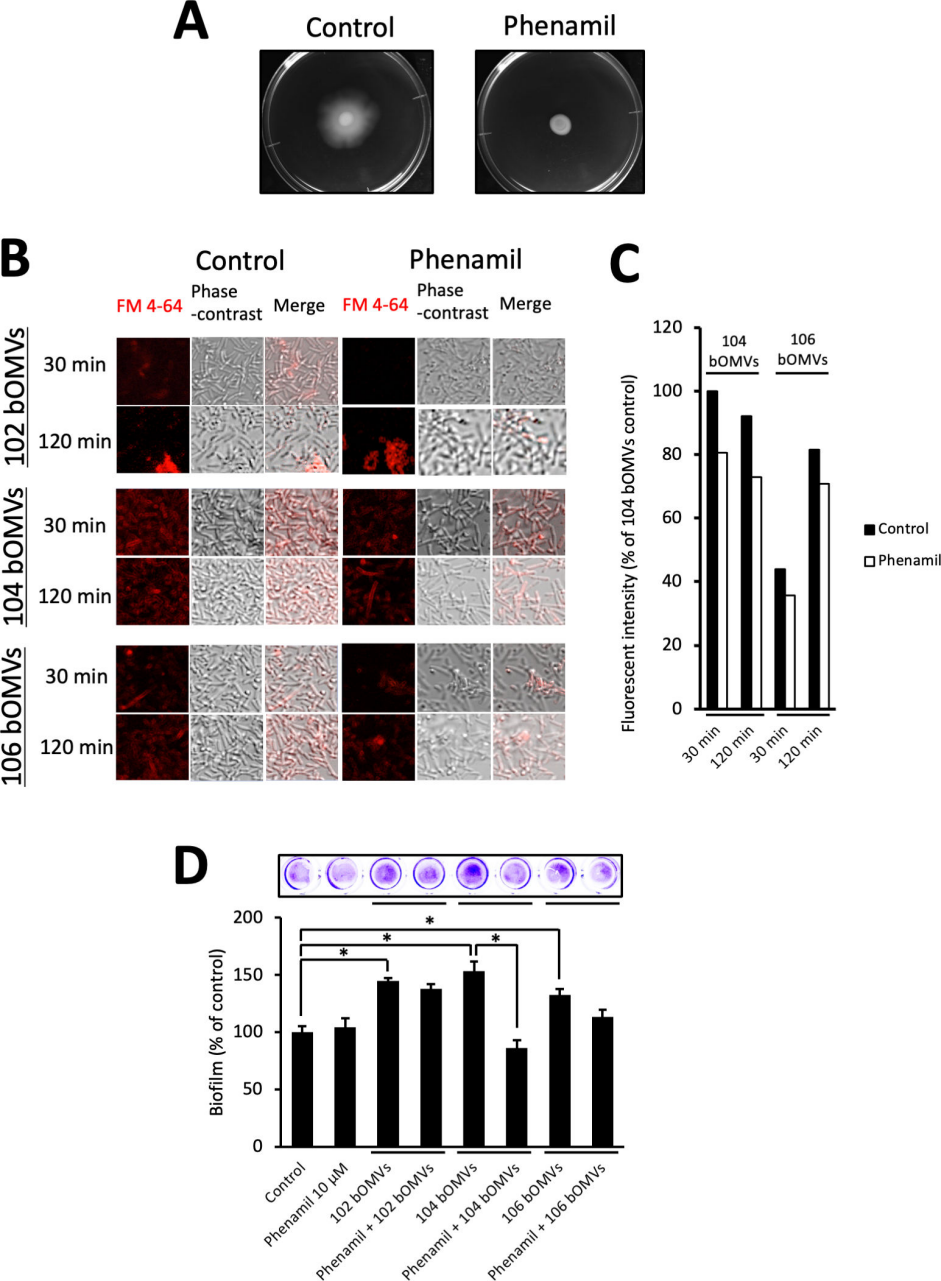
Flagellar motility influences the interaction between bacterial cells and bOMVs through multiple mechanisms. (**A**) Observation of flagellar-mediated bacterial migration of strain 106 (control, left panel). The addition of 10 µM phenamil completely inhibited flagellar-mediated bacterial migration (10 µM phenamil, right panel). (**B**) Strain 106 was incubated with FM 4-64-labeled 102 bOMVs, 104 bOMVs, and 106 bOMVs at 30°C for 30 or 120 min in the absence (panels in the column labeled “control”) or presence of 10 µM phenamil (panels in the column labeled “10 µM phenamil”). The panels in the “FM 4-64” column depict the results observed with confocal laser microscopy. The panels in the “phase-contrast” column show the results of observation under a phase contrast microscope. The “merge” column shows a superposition of the results observed with the confocal laser microscope and the phase contrast microscope. (**C**) The fluorescence intensity in the experiment using 104 bOMVs and 106 bOMVs in panel **B** was measured and shown as a graph. (**D**) Comparison of the biofilm-forming ability of strain 106 incubated with purified 102 bOMVs, 104 bOMVs, and 106 bOMVs in the absence or presence of 10 µM phenamil. Biofilm-forming ability was assessed using the crystal violet method (photograph of the microplate at the top of the figure) as previously reported (4). The biofilm-forming ability of strain 106 in the absence of both purified bOMVs and 10 µM phenamil was set as 100%. Data are shown as the mean and standard error. * indicates a significant difference at *P* < 0.05.

The results shown in Fig. 6 indicate that the extent of interaction of 106 Fla may vary, depending on the type of bOMVs involved. To examine whether flagella motility contributes to the differences in the interactions between strain 106 and the various bOMVs, we performed experiments using phenamil, which inhibits the sodium channel required for flagellar motility. As shown in Fig. 7A, the addition of 10 µM phenamil inhibited the migration ability of strain 106 on the agar medium. After the lipid components of bOMVs from the three strains were labeled with a fluorescent substance (FM 4-64), their interactions with strain 106 were observed by confocal laser microscopy in the absence or presence of 10 µM phenamil (Fig. 7B).

As a result, the quantity of FM 4-64-labeled bOMVs from strains 104 (FM-104 bOMVs) and 106 (FM-106 bOMVs) attached to the bacteria was reduced in the presence of 10 µM phenamil, suggesting that inhibition of flagellar movement reduces the adhesion of 104 or 106 bOMVs to the bacterial cells (Fig. 7B, 104 bOMVs and 106 bOMVs, Fig. 7C). However, the quantity of FM 4-64‒labeled bOMVs from strain 102 (FM-102 bOMVs) attached to the bacteria exhibited only a slight decrease even upon the inhibition of flagellar motility by 10 µM phenamil (Fig. 7B, 102 bOMVs). Thus, it was assumed that the way of bOMV attachment to the bacterial cells appeared to vary, depending on the bOMVs used.

Furthermore, we assessed the biofilm-forming ability of strain 106 when exposed to different bOMVs in the absence or presence of 10 µM phenamil. As shown in Fig. 7D, the biofilm-forming ability of strain 106 in the presence of purified 104 bOMVs was significantly reduced by the addition of 10 µM phenamil. A decreasing trend was also noted in the presence of 106 bOMVs, although the variation was not significant. However, the enhancement of biofilm formation by strain 106 in the presence of purified 102 bOMVs was only minimally affected by the addition of 10 µM phenamil.

## DISCUSSION

Previously, we reported the results concerning biofilm formation in *Aeromonas* spp. (4). In that report, we showed that bOMVs, which are OMVs derived from biofilms, play a role in promoting biofilm formation (4). However, we found that the ability of *Aeromonas* spp. to form biofilms differs, depending on the strain (4). A typical example in *A. veronii* sobria is shown in Fig. 1; that is, the biofilm-forming ability of strain 106 was found to be higher than that of strain 104, which in turn was higher than that of strain 102. This result suggests the potential presence of a factor impacting the biofilm-forming ability of *A. veronii* sobria strains. Since it was also presumed that the factors may affect the biofilm formation-promoting effect of bOMVs, we first examined the effect of bOMVs biofilm formation in these three strains of *A. veronii* sobria. We found that the strain 106 with the strongest biofilm-forming ability and susceptibility to enhanced biofilm formation by bOMVs had flagella and that these flagella promoted the interaction between bOMVs and bacteria. In addition, based on the protein analysis shown in Table 1, it was considered to be polar flagella because the protein analysis showed that the detected protein was Fla, which constitutes the polar flagellum. Although it has been widely known that flagella are involved in bacterial motility and adhesion to targets (19, 23), the findings of this study may lead to a novel function of flagella in the interaction between bacteria and bOMVs. To understand the role of flagella in the interaction between bacteria and bOMVs, we performed observation using TEM.

TEM observations illustrated in Fig. 6 revealed that 106 Fla bound with bOMVs originating from each strain across multiple observation fields. However, the interaction between the flagella and each bOMV seemed to vary based on the strain of origin. Specifically, 102 bOMVs exhibited a propensity for more facile interaction in the presence of 106 Fla compared to bOMVs obtained from other strains, with a tendency for 102 bOMVs to aggregate with each other (Fig. 6B). In contrast, it was observed that 104 bOMVs interacted less frequently with 106 Fla (Fig. 6C), whereas 106 bOMVs seemed to interact with 106 Fla at some frequency (Fig. 6D). As discussed above, these observations indicate that the characteristics of bOMVs vary, depending on the originating strain. The physical properties of OMVs have been reported through phase imaging using high-speed atomic force microscopy, revealing distinct adsorption and viscoelastic properties among individual particles (24). The present results suggest that OMVs released from even the same bacterial strain constitute a heterogeneous population of particles with diverse physical characteristics; bOMVs binding to 106 Fla, as shown in Fig. 6, are presumably more inclined to do so with highly adsorptive bOMVs. However, since it is known that *Aeromonas* also expresses lateral flagella under certain conditions, the effects of lateral flagella may also need to be investigated in the future.

On the other hand, it has been reported that the bOMVs originate from cell lysis, and explosive cell lysis will not only give rise to vesicles but also concomitantly lead to the release of eDNA, which is an important biofilm matrix material (25, 26). As shown in Fig. S2B, eDNA was confirmed by agarose gel electrophoresis in the ECM fractions, but eDNA was not detected in the purified bOMV fractions. We also attempted to detect DNA using the PicoGreen assay because DNA detection using agarose gel has low sensitivity. As a result, we were able to confirm the presence of trace amounts of DNA in the bOMV samples, but we were not able to reach a sufficient conclusion regarding the amount of eDNA contained in bOMVs in this study. We think that further analysis is needed to determine the relationship between the interaction between bOMVs and flagella and the eDNA contained in bOMVs. In general, the association of OMVs with bacteria is described to involve hydrophobic or electrostatic interactions (27). Another study has also indicated that OMVs are more hydrophobic than the bacterial surface and that the physical traits of OMVs adhering to the bacterial surface undergo alterations over time (28), thereby proposing that not all OMVs engage with the flagella. This dynamic could potentially explain why certain OMVs bind to the flagella, as depicted in Fig. 6.

It has been reported that the adhesion ability of *Vibrio alginolyticus* is reduced by inhibiting sodium-driven flagellar motility (19). Since *Aeromonas* is a bacterium belonging to the Vibrionaceae family, we carried out experiments using phenamil, an inhibitor of sodium ion-driven flagellar motor rotation. As shown in Fig. 7A, the addition of 10 µM phenamil caused inhibition of bacterial migration of 106 strain on agar medium, suggesting that *A. veronii* sobria also exhibits sodium-driven flagellar motility. We performed additional experiments to compare the process from bacterial adhesion to biofilm formation on microplates using strains 106 and *V. parahaemolyticus* RIMD 2210633 (Fig. S3). First, we observed the migration of strain 106 and *V. parahaemolyticus* on lysogenic broth (LB) agar medium (Fig. S3A) and quantified the diameter of the migration zone (Fig. S3B). As shown in these results, the addition of 10 µM phenamil sufficiently inhibited the bacterial migration of strain 106 (same as shown in Fig. 7A) but not of *V. parahaemolyticus*. The addition of 100 µM phenamil sufficiently inhibited the bacterial migration of *V. parahaemolyticus*. Under these conditions, the adhesion of bacteria to microplates was compared in terms of the degree of biofilm formation. The results showed that the addition of phenamil had little effect on strain 106, whereas the addition of phenamil significantly reduced the degree of adhesion of *V. parahaemolyticus* (Fig. S3C). Based on these results, we think that inhibition of flagellar motility in *V. parahaemolyticus* significantly reduces not only motility but also biofilm formation as observed in *V. alginolyticus* (19), whereas inhibition of flagellar motility of strain 106 does not affect biofilm formation. We therefore presume that, even though the flagella of strain 106 have lost motility, the strain can still form biofilms.

Even though they are all referred to as flagella, variations exist, including those with or without sheaths. For example, it has been reported that *Vibrio* spp. (29, 30) and *H. pylori* (31) possess flagellum having a sheath composed of bacterial outer membrane components on the outside of the flagellum. Several reports have also revealed that the outer membrane composition differs from the lipid composition and protein orientation of the bacterial outer membrane (32). On the other hand, it has been reported that *E. coli* flagella, which do not have sheaths, are involved in bacterial adhesion to abiotic surfaces (33) and to the host protein, TLR5 (34). Although the physiological function of the flagellar sheath is unclear, it is quite possible that the presence or absence of the flagellar sheath affects interactions with external structures such as bOMVs. Thus, further analysis is indispensable for elucidating the molecular-level interaction between *A. veronii* sobria flagella and bOMVs to clarify our above idea.

As shown in Fig. 6, the state of interaction between bOMVs and flagella seemed to vary among bOMVs from different strains. It is necessary to consider the overall impact of this phenomenon on the bacteria and OMVs. Therefore, we fluorescently labeled purified bOMVs with FM 4-64 and analyzed their interactions with *A. veronii* sobria strain 106, which possesses flagella. The results showed the following: FM-102 bOMVs tended to bind less to bacterial cells than the other bOMVs (Fig. 7B; 102 bOMVs and control). On the other hand, FM 4-64 fluorescence was observed closer to the bacterial cells, with no significant change in the presence of 10 µM phenamil (Fig. 7B; 102 bOMVs and 10 µM phenamil). Taken together, these results and those in Fig. 6B suggest that 102 bOMVs have a high affinity for flagella, predominantly binding to them more rapidly than they interact with the bacterial cell surface. Thus, the 102 bOMVs appeared to be aggregated locally. Since adhesion proceeds when 102 bOMVs encounter flagella, even when flagellar motility is inhibited, it is likely that local assembly of 102 bOMVs occurred even in the presence of 10 µM phenamil. This hypothesis is supported by the fact that bacterial biofilm formation by *A. veronii* sobria strain 106 in the presence of purified 102 bOMVs was not affected even in the presence of 10 µM phenamil (Fig. 7C). However, due to potential variations in staining effectiveness using FM4-64 among different bOMVs, there is a possibility that even if 102 bOMVs do attach to bacterial cells, they might not be identifiable through visualization. Although the mechanism by which 102 bOMVs promote biofilm formation in strain 106 is currently unknown, studies on *Bacillus subtilis* have indicated that physical stress on flagella stimulates adhesin expression, enhancing bacterial adhesion (35, 36). In other words, the strong binding of 102 bOMVs to 106 Fla, as shown in Fig. 6B, may be a physical stress that promotes adhesin production, thereby enhancing biofilm formation.

In contrast, FM-104 bOMVs seemed to be uniformly rather than locally attached to the surface of all bacterial cells (Fig. 7B, 104 bOMVs and control). The adhesion of FM-104 bOMVs to individual bacterial cells was somewhat reduced in the presence of 10 µM phenamil (Fig. 7B, 104 bOMVs and 10 µM phenamil). Accordingly, biofilm formation by *A. veronii* sobria strain 106 in the presence of purified 104 bOMVs was significantly reduced by the addition of 10 µM phenamil (Fig. 7C). Considering Fig. 6C, these phenomena may be attributable to the characteristic of 104 bOMVs exhibiting a stronger attraction toward the bacterial cell surface rather than flagella. As bacteria are propelled by flagellar action, increased numbers of bacteria and 104 bOMVs encounter each other, and numerous 104 bOMVs adhere to the bacterial cell surface, potentially enhancing biofilm formation. However, we have not obtained any physical evidence to support our hypothesis above at present. We therefore need to conduct detailed molecular-level analysis of the interactions between bOMVs and flagella or cell surface components.

FM-106 bOMVs appeared to be attached not only to the bacterial surface but also to localized areas of some bacteria (Fig. 7B, 106 bOMVs and control). In addition, the adhesion of FM-106 bOMVs to the bacterial cell surface was reduced in the presence of 10 µM phenamil but still occurred locally (Fig. 7B, 106 bOMVs and 10 µM phenamil). These results suggest that the 106 bOMVs may possess traits of both 102 bOMVs and 104 bOMVs as described above. Concomitantly, biofilm formation by strain 106 in the presence of purified 106 bOMVs tended to decrease with the addition of 10 µM phenamil, but the difference was not significant (Fig. 7C). This result suggests that even if flagellar motility is inhibited by 10 µM phenamil, interactions persist between 106 bOMVs and flagella, akin to interactions observed between 102 bOMVs and flagella. These interactions could potentially contribute to a certain level of biofilm formation. We therefore think that the 106 bOMVs bind to both the bacterial surface and flagella when strain 106 is cultured in the presence of 106 bOMVs.

Furthermore, it may be also necessary to consider whether the interaction between bOMVs and bacterial cells activates additional signal transduction systems within the cells, inducing physiological changes in the bacteria, including biofilm formation. In *Pseudomonas aeruginosa*, which typically forms biofilms, it has been reported that fluctuations in c-di-GMP within the cells ultimately have a major influence on biofilm formation (37–39). Hence, we believe that these alterations in bacteria’s physiological functions necessitate comprehensive analysis. We therefore must plan to conduct further research into how physiological changes, including changes in the signal transduction system within bacterial cells, transpire as a result of the interaction between bOMVs and bacterial cells, leading to biofilm formation.

In this study, we discovered that a certain type of bOMV released from the bacteria preferentially interacts with bacterial flagella. In conclusion, we propose that increased interactions between bOMVs and bacterial cells (bacterial cell surface and bacterial flagella) may promote bacterial adhesion and subsequent biofilm formation. To verify our idea, it is necessary to investigate the physical properties of bOMVs in detail in the future to clarify which physical properties of bOMVs cause differences in biofilm formation.

## MATERIALS AND METHODS

### Bacterial strains and culture media used in this study

Three *A. veronii* sobria clinical strains (102, 104, and 106) were used in this study. These strains were employed in our previous studies (4, 40, 41). *A. veronii* sobria strains (102, 104, and 106) were cultured overnight at 37°C on lysogeny broth (LB) agar medium. Colonies were then selected, transferred to LB medium, and incubated overnight at 37°C for subsequent analyses. LB agar and LB broth were purchased from Becton Dickinson (Tokyo, Japan).

### Biofilm formation of *A. veronii* sobria strains used in this study

Biofilm formation on 96-well flat-bottom polystyrene plates (Corning, Glendale, AZ, USA) was examined using the *A. veronii* sobria strains (102, 104, and 106) cultured in LB medium at 30°C for 18 h according to our previous method (4). In previous studies, bOMVs were collected after 24 h of culture, but the properties of the bOMVs obtained after 18 h of culture were not significantly different from those obtained after 24 h of culture in promoting biofilm formation. We therefore set the culture time at 18 h for operational efficiency. Experiments were performed in triplicate on two independent occasions.

### Effect of bOMVs on *Aeromonas* biofilm formation

The *A. veronii* sobria strains (102, 104, and 106) were cultured in LB medium at 37°C for 18 h. Subsequently, the overnight cultures were resuspended in Hanks’ balanced salt solution (+) buffer and transferred to 96-well flat-bottom polystyrene plates (Corning). Purified bOMVs (the purification method for bOMVs is described below) were then added to the bacteria, followed by co-incubation at 30°C for 2 h. The biofilms formed on the polystyrene plates were washed twice with PBS (FUJIFILM Wako Pure Chemical, Osaka, Japan) and fixed with 4% paraformaldehyde in PBS at room temperature. The biofilms were further washed twice with PBS. After removal of the supernatants, the biofilms were stained with 5% crystal violet for 10 min, followed by another cycle of PBS washing. Then, 100 µL of a decolorizing agent (70% ethanol and 30% acetone) was introduced to extract the crystal violet dye from the stained biofilms. The absorbance of crystal violet within the destained solution was measured at 595 nm using an optical microtiter plate reader (Molecular Devices, Sunnyvale, CA, USA).

### Purification of bOMVs

The bOMVs were purified according to previous methods (4, 42, 43). Briefly, the *A. veronii* sobria strains (102, 104, and 106) were cultured on LB agar plates at 30°C for 18 h. The bacterial biofilms were then suspended in sterile PBS and centrifuged at 10,000 × *g* for 30 min at 4°C. The supernatant was passed through a 0.22 µm membrane filter (NIPPON Genetics, Tokyo, Japan), and particulate components in the solution were harvested by ultracentrifugation (100,000 × *g* for 2 h at 4°C) using a P65ST rotor (Hitachi, Tokyo, Japan). The pellet was washed twice with PBS and resuspended in sterile PBS containing 30% (vol/vol) iodixanol (OptiPrep; Sigma-Aldrich, St. Louis, MO, USA) (0.9 mL). The components in the suspension were fractionated on OptiPrep density gradient centrifugation using 10% (0.9 mL), 15% (0.9 mL), 20% (0.9 mL), 25% (0.9 mL), and 30% (0.9 mL) (vol/vol) iodixanol solutions. The samples were then ultracentrifuged to equilibrium (100,000 × *g* for 17 h at 4°C) using a P65ST rotor and fractionated into 450 µL aliquots. After each fraction was analyzed by SDS-PAGE, fractions containing bOMVs were concentrated and washed via ultracentrifugation and then purified. The protein concentration of each bOMV in each sample was assessed using the Bradford method (Bio-Rad Laboratories, Hercules, CA, USA). The presence of the bOMVs in the fractionated samples was confirmed through observation using a TEM (detailed below). The sample containing purified bOMVs was stored at −80°C until use. bOMVs from strains 102, 104, and 106 bOMVs of *A. veronii* sobria were individually purified from their respective biofilms.

### Electron microscope observation (TEM and SEM)

The verification of the purified bOMVs involved observation using a TEM. The bOMV preparations were affixed onto a carbon-coated copper grid (400 mesh) and subjected to negative staining with 2% (wt/vol) uranyl acetate. In addition, SEM was employed to scrutinize the surface structures of the *A. veronii* sobria strains. Both the TEM and SEM examinations were conducted at the Hanaichi Electron Microscope Technical Laboratory (Aichi, Japan).

### Identifying proteins of the flagella-like structures

While purifying bOMVs from *A. veronii* sobria strain 106, unique proteins not seen in the other strains were observed. When the fraction containing the proteins was observed under SEM and TEM, flagella-like structures were confirmed. These structures were then isolated and subjected once more to SDS-PAGE. The gel was then stained using a silver stain MS kit (Nacalai Tesque, Kyoto, Japan). The two major protein bands (sample 1 and sample 2) detected from the electrophoretic gel were excised, and these gel portions were dispatched to Japan Proteomics (Miyagi, Japan) to identify the proteins contained in the gels. The proteins in each excised gel were characterized through nano-LCMS/MS analysis.

### Interaction of bOMVs with flagella

Flagella purified from the *A. veronii* sobria strain 106 (5 μg/mL) and bOMVs from different strains (5 µg/mL) were combined and incubated in PBS at 30°C for 2 h. The interaction between the two was then observed by TEM.

### Swimming motility

Swimming is defined as flagellum-directed movement in an aqueous environment. To investigate the effects of flagellar motility inhibition, we used phenamil (Tocris Bioscience, Ellisville, MO, USA), a compound that inhibits flagella-driven bacterial migration (44). To examine swimming motility, 5 µL of overnight cultures was deposited at the center of both a 0.3% LB agar plate and a 0.3% LB agar plate containing 10 µM phenamil, followed by a 16 h incubation at 30°C, after which the plates were photographed.

### Statistical analysis

The data presented are the averages of three samples. Error bars represent standard deviations. For multiple comparisons, one-way analysis of variance (ANOVA) was used, followed by Tukey’s test (*P < 0.05) in Fig. 7D and Dunnett’s test (*P < 0.05) in Fig. 1, 2A through F and 6C. The statistical analyses were carried out using Easy R (Saitama Medical Center, Jichi Medical University) (45).

## SUPPLEMENTAL MATERIAL

Supplemental figures spectrum.02838-24-s0001.pdf Fig. S1 to S3.

Supplemental material spectrum.02838-24-s0002.docx Supplemental figure legends.

ASM does not own the copyrights to Supplemental Material that may be linked to, or accessed through, an article. The authors have granted ASM a non-exclusive, world-wide license to publish the Supplemental Material files. Please contact the corresponding author directly for reuse.
