# Supplementary figures and images for: Flagella of Aeromonas veronii biotype sobria promote biofilm formation by biofilm-derived outer membrane vesicles (bOMVs)
Source: Microbiol Spectr. 2025 Oct 27;13(12):e02838-24. doi: 10.1128/spectrum.02838-24 (PMC12671187; doi:10.1128/spectrum.02838-24)

106 Fla

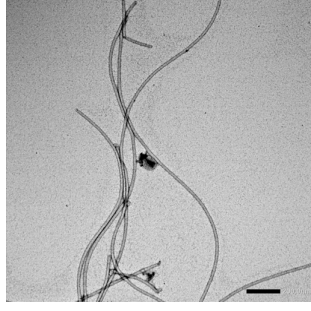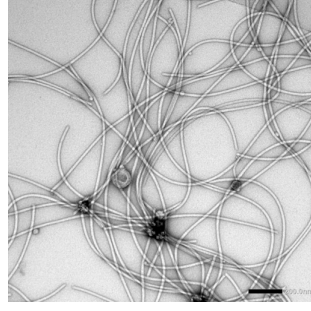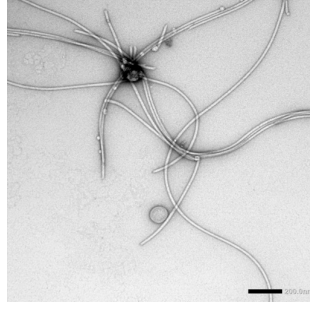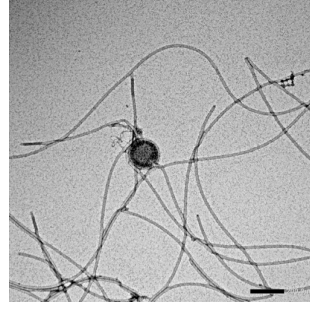

106 Fla-102 bOMVs

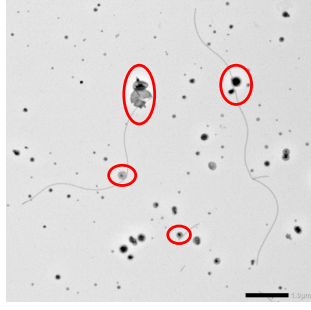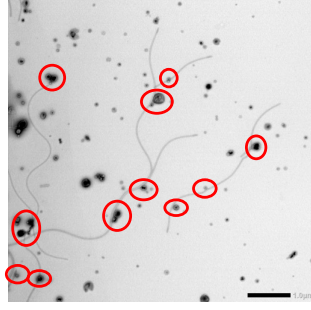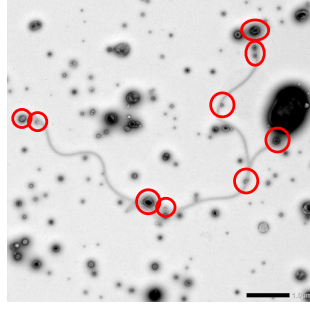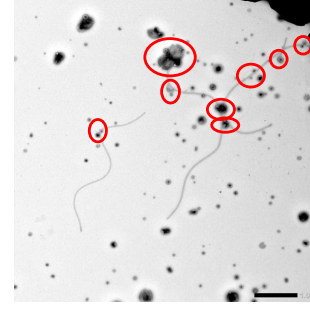

106 Fla-104 bOMVs

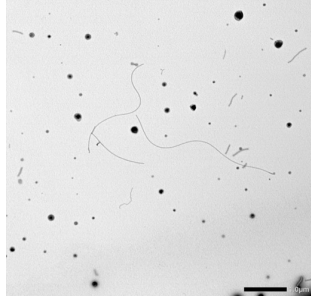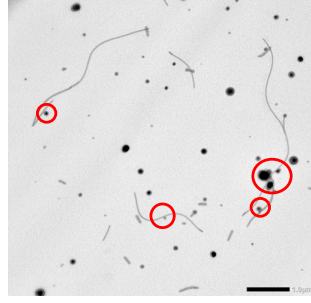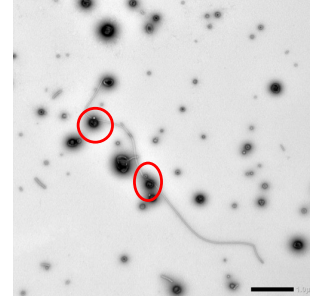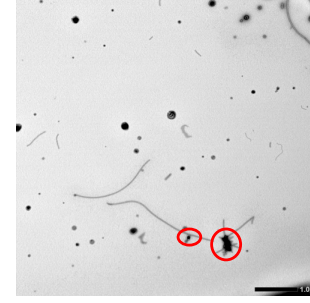

106 Fla-106 bOMVs

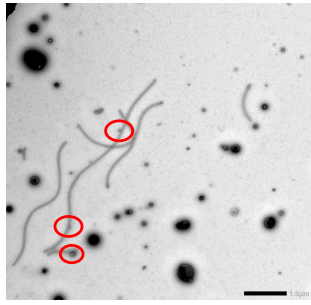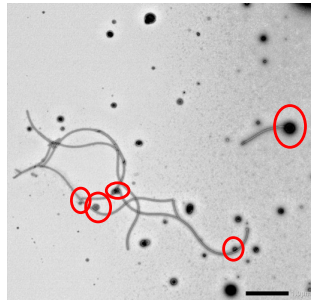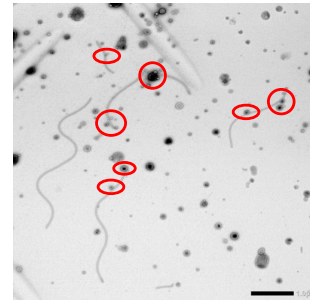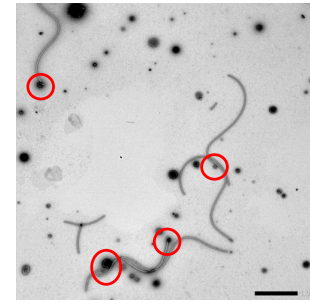

# A

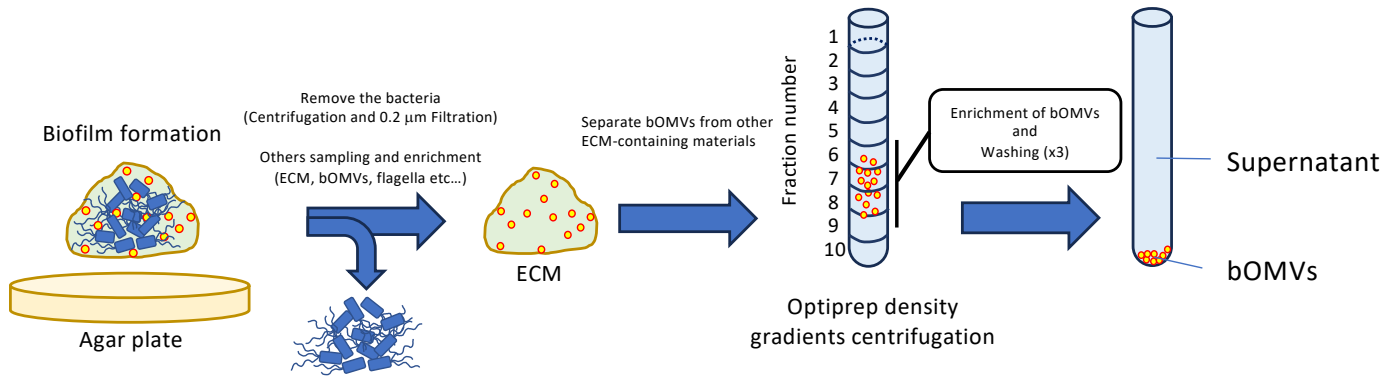

# B

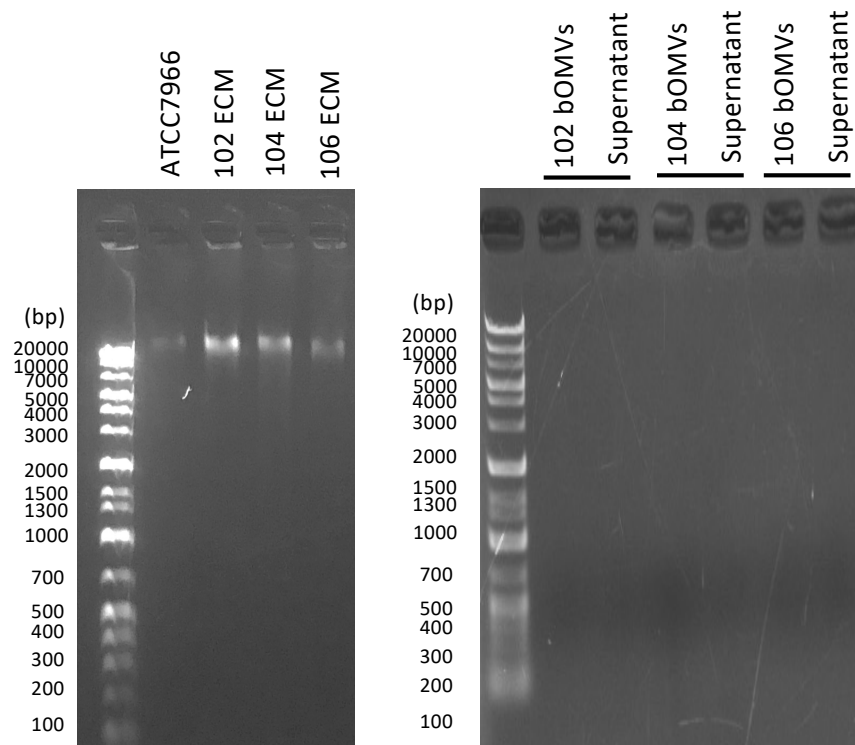

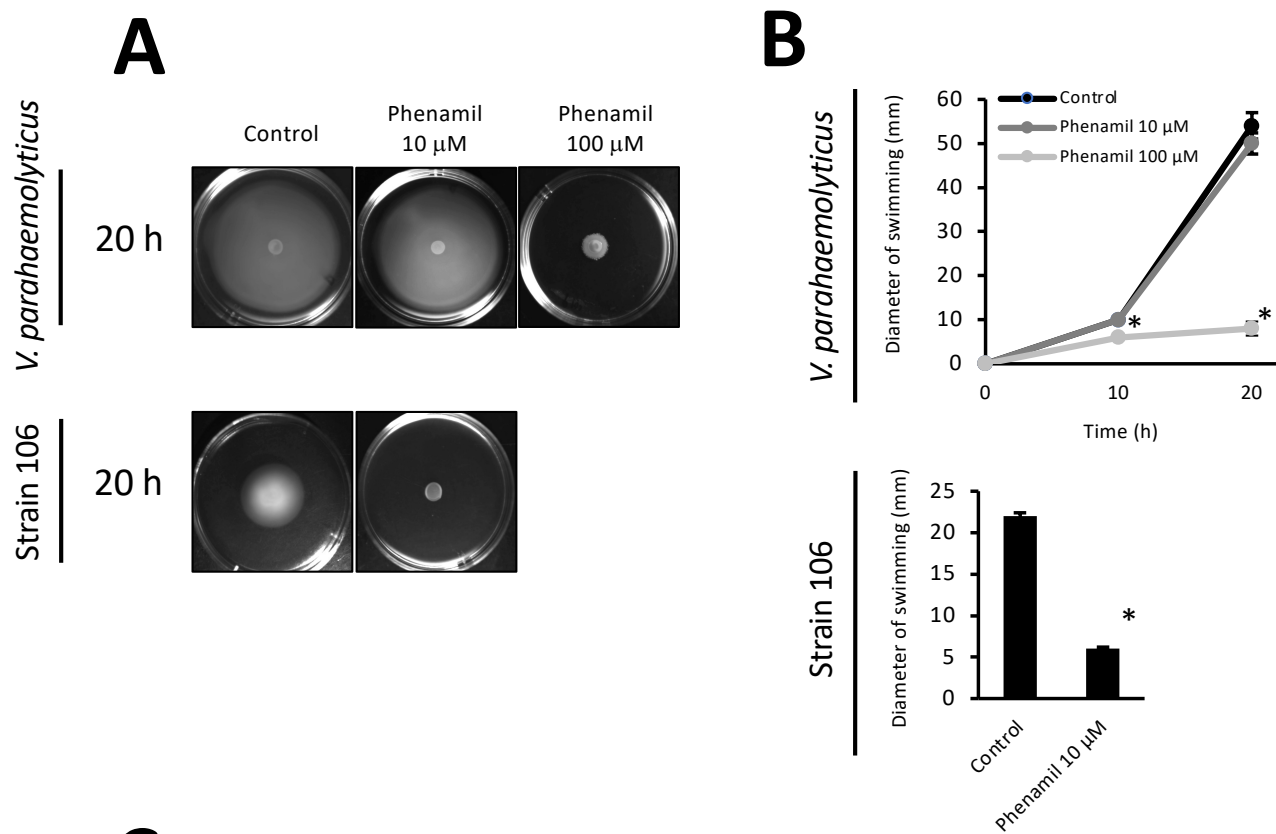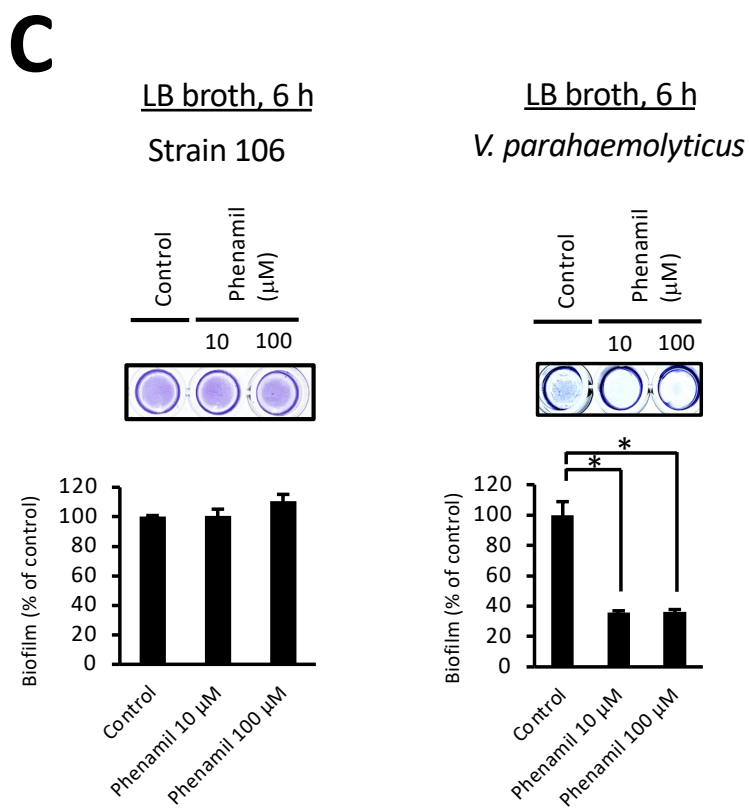

Supplement: Supplemental figures — Fig. S1 to S3. [file spectrum.02838-24-s0001.pdf]
