# Supplementary material for: Flagella of Aeromonas veronii biotype sobria promote biofilm formation by biofilm-derived outer membrane vesicles (bOMVs)
Source: Microbiol Spectr. 2025 Oct 27;13(12):e02838-24. doi: 10.1128/spectrum.02838-24 (PMC12671187; doi:10.1128/spectrum.02838-24)
Supplement: Supplemental material — Supplemental figure legends. [file spectrum.02838-24-s0002.docx]

**Supplemental figure legends**

**Fig. S1. Quantification of the total number of bOMVs bound to flagella.** To quantify the TEM observation shown in Figs. 6 A to D, we analyzed TEM images taken from four fields (Images 1 to 4) at a low-magnification (x 5,000). We quantified the number of bOMVs associated with flagella in four fields of view. Red circles indicate points of interaction between flagella and various bOMVs.

**Fig. S2. Detection of eDNA in purified bOMVs.** (A) Schematic diagram of the purification of bOMVs by Optiprep density gradient centrifugation. ECM fractions of *A. veronii* sobria strains 102, 104 and 106 were prepared. bOMVs were purified from these preparations by Optiprep density gradient centrifugation. (B) ECM fractions of *A. veronii* sobria strains 102, 104 and 106 (left side panel), as well as purified bOMVs and their supernatant fractions, were subjected to agarose gel electrophoresis to detect eDNA (right side panel).

**Fig. S3. Effect of phenamil, an inhibitor of sodium ion-driven flagellar motor rotation, on adhesion of *A. veronii* sobria strain 106 or *V. parahaemolyticus* RIMD2210633 strain to microplates.** (A) Effect of phenamil on bacterial migration on LB agar medium. (B) Measurement of the diameter of the bacterial migration zone on LB agar medium. (C) After culturing the bacteria in LB medium, adhesion of bacteria to microplates in the presence or absence of phenamil was compared in terms of the extent of biofilm formed after addition of bacteria to the microplates. Experiments were performed in triplicate on two independent occasions. Data are shown as the mean and standard error. Data are shown as the mean and standard error. * indicates a significant difference at *p*<0.05.
